# Patterns of Coral Disease across the Hawaiian Archipelago: Relating Disease to Environment

**DOI:** 10.1371/journal.pone.0020370

**Published:** 2011-05-31

**Authors:** Greta S. Aeby, Gareth J. Williams, Erik C. Franklin, Jean Kenyon, Evelyn F. Cox, Steve Coles, Thierry M. Work

**Affiliations:** 1 Hawai'i Institute of Marine Biology, University of Hawaii, Kaneohe, Hawai'i, United States of America; 2 School of Biological Sciences, Victoria University of Wellington, Wellington, New Zealand; 3 Center for Marine Biodiversity and Conservation, Scripps Institution of Oceanography, La Jolla, California, United States of America; 4 Joint Institute for Marine and Atmospheric Research, University of Hawai'i, and NOAA Pacific Islands Fisheries Science Center, Honolulu, Hawai'i, United States of America; 5 University of Hawai'i — West Oahu, Pearl City, Hawai'i, United States of America; 6 Hawai'i Institute of Marine Biology, Kane'ohe, Hawai'i, United States of America; 7 Bishop Museum, Honolulu, Hawai'i, United States of America; 8 U. S. Geological Survey, National Wildlife Health Center, Honolulu Field Station, Honolulu, Hawai'i, United States of America; King Abdullah University of Science and Technology, Saudi Arabia

## Abstract

In Hawaii, coral reefs occur across a gradient of biological (host abundance), climatic (sea surface temperature anomalies) and anthropogenic conditions from the human-impacted reefs of the main Hawaiian Islands (MHI) to the pristine reefs of the northwestern Hawaiian Islands (NWHI). Coral disease surveys were conducted at 142 sites from across the Archipelago and disease patterns examined. Twelve diseases were recorded from three coral genera (*Porites, Montipora, Acropora*) with *Porites* having the highest prevalence. *Porites* growth anomalies (PorGAs) were significantly more prevalent within and indicative of reefs in the MHI and *Porites* trematodiasis (PorTrm) was significantly more prevalent within and indicative of reefs in the NWHI. *Porites* tissue loss syndrome (PorTLS) was also important in driving regional differences but that relationship was less clear. These results highlight the importance of understanding disease ecology when interpreting patterns of disease occurrence. PorTrm is caused by a parasitic flatworm that utilizes multiple hosts during its life cycle (fish, mollusk and coral). All three hosts must be present for the disease to occur and higher host abundance leads to higher disease prevalence. Thus, a high prevalence of PorTrm on Hawaiian reefs would be an indicator of a healthy coral reef ecosystem. In contrast, the high occurrence of PorGAs within the MHI suggests that PorGAs are related, directly or indirectly, to some environmental co-factor associated with increased human population sizes. Focusing on the three indicator diseases (PorGAs, PorTrm, PorTLS) we used statistical modeling to examine the underlying associations between disease prevalence and 14 different predictor variables (biotic and abiotic). All three diseases showed positive associations with host abundance and negative associations with thermal stress. The association with human population density differed among disease states with PorGAs showing a positive and PorTrm showing a negative association, but no significant explanatory power was offered for PorTLS.

## Introduction

Coral disease has emerged as a serious threat to coral reefs worldwide and a major cause of reef deterioration [1]. The numbers of diseases, coral species affected, and the distribution of diseases have all increased dramatically within the last decade [2], [3], [4], [5]. Epizootics of coral disease have resulted in significant losses of coral cover. An outbreak of white band disease in the 1980s killed acroporid corals throughout the Caribbean [6], [7], and a recent outbreak of white pox disease in the Florida Keys reduced the cover of *Acropora palmata* by up to 70% [8]. In the Caribbean, coral disease has been implicated as a major factor contributing to the catastrophic decline of coral reefs, resulting in an apparent ecological phase shift from coral to algal-dominated ecosystems [2], [4], [6], [9]. Disease is now emerging as a problem in the Indo-Pacific. The Australian Institute of Marine Science Long Term Monitoring Program (AIMS LTMP) documented a 22- to 150-fold increase in white syndrome between 1998 and 2003 on the Great Barrier Reef [10]. Coral disease has also been reported from the Philippines [11], [12], Indonesia [13], the Marshall Islands and Palau [14], American Samoa [15], [16], and the US remote Pacific Island areas [17], [18], [19].

Disease emerges from a complex interplay between host, agent and environment [20],[21],[22],[23]. Disease prevalence and distribution depends on host distribution, abundance and environmental cofactors [20], [21], [22], [24]. A basic premise of epidemiology is that increased host abundance enhances introduction and maintenance of infectious disease [25], [26], [27]. Coral disease is no exception with numerous studies finding a relationship between disease prevalence and host abundance [28], [29], [30], [31], [32]. Increased anthropogenic stress on nearshore environments, overfishing, and environmental conditions associated with global climate change have all been implicated as contributing to increased coral disease [20], [21], [24]. For example, increases in black-band disease were observed by Antonius [33] in polluted waters near industrialized areas and by Bruckner et al. [34] in areas where high sedimentation and algal overgrowth prevailed. Raymundo et al. [35] found that marine protected areas had higher fish diversity and lower disease prevalence than overfished reefs. Bruno et al. [28] reported that outbreaks of white syndrome in scleractinian corals on the GBR were not only correlated with host abundance but also with warm sea surface temperature anomalies. Increasing local human pressures combined with environmental changes associated with global climate change place coral reefs, worldwide, at risk for collapse. If we are to maintain our coral reef resources then a better understanding is needed of environmental cofactors in occurrence of disease.

Hawaii, is in the unique position of having coral reefs which occur across a wide gradient of biological (host abundance), climatic (sea surface temperature anomalies) and anthropogenic conditions from the heavily human-impacted reefs of the eight, main Hawaiian Islands (MHI) to the relatively pristine reefs of the northwestern Hawaiian Islands (NWHI). The MHI are severely overfished compared to the NWHI [36] and suffer from chronic problems such as coastal development and subsequent terrestrial runoff, coastal pollution from injection wells and sewage spills and human activities associated with tourism and marine recreation [37]. Thus, the Hawaiian archipelago can serve as a ‘natural’ experiment to examine which factors may be important in influencing patterns of coral disease in the field. Our objectives were to: 1) document the types, frequency of occurrence and prevalence of coral disease across the Hawaiian archipelago, 2) determine whether there were any genus level differences in disease susceptibility in Hawaii, 3) examine whether patterns of disease occurrence differ between regions (MHI vs. NWHI) or within regions (among islands), and 4) focusing on three indicator diseases, use statistical modeling to explore underlying associations between disease prevalence and 14 different predictor variables (biotic and abiotic) that could affect disease processes or help explain presence of disease.

## Methods

### Study area

**Figure 1:**
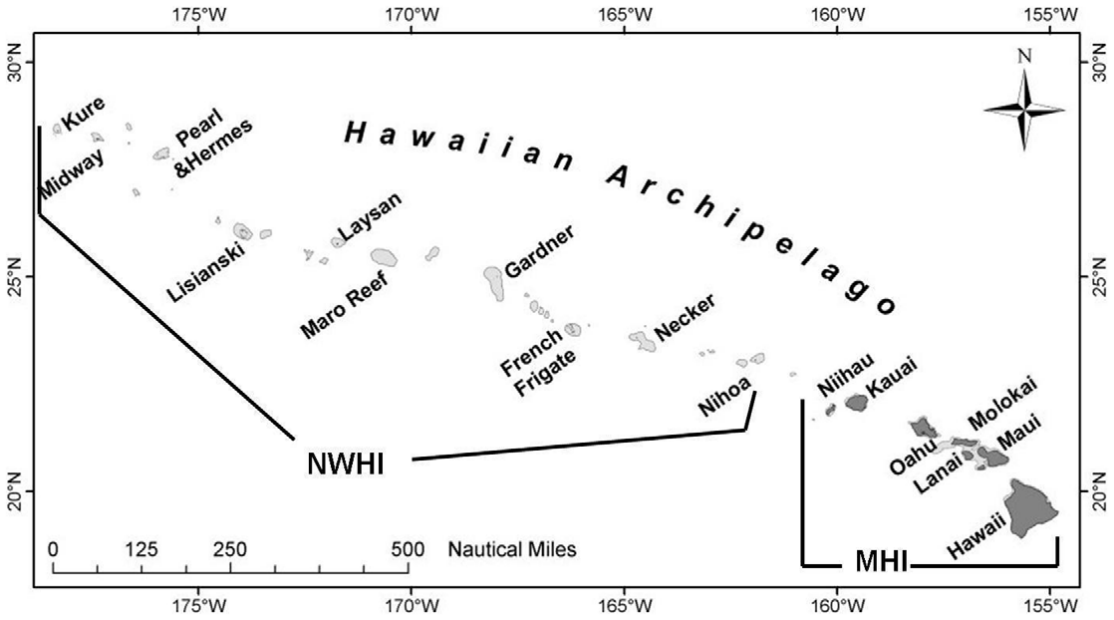
Map of the Hawaiian archipelago.

The Hawaiian archipelago is one of the most isolated archipelagos in the world spanning over 2,500 km from the island of Hawai'i in the southeast to Kure Atoll in the northwest (Figure 1). It is composed of two regions: the populated eight main Hawaiian Islands (MHI) and the mostly uninhabited islands, atolls and banks of the Northwestern Hawaiian Islands (NWHI). The MHI consists of high volcanic islands with non-structural reef communities and fringing reefs abutting the shore. Approximately 1.2 million people live in the MHI with another nearly seven million tourists visiting Hawaii each year [37]. This large population of residents and visitors has affected the coral reefs of Hawaii through urban development, land-based sources of pollution, overfishing, and recreational overuse. In contrast, the majority of the islands, shoals and atolls within the NWHI are uninhabited, except for Midway, which has been continuously occupied by a limited number of people since 1908, and Kure, Laysan Island, French Frigate Shoals and recently Lisianski, which have been intermittently occupied over the last century. The remoteness and limited reef fishing activities in the NWHI have resulted in significantly reduced anthropogenic impacts to this region as compared to the MHI [37].

### Disease surveys

Surveys were conducted at 78 sites from six different islands within the MHI in 2004 (May, June) and 2005 (February, June, July) and 64 sites from eight different islands/atolls in the NWHI in 2004 (September and October) (Table 1). These surveys were conducted as part of larger multi-agency studies obtaining baseline information on coral reefs throughout the Hawaiian archipelago and so were constrained as to location and timing of the surveys. At each site, two consecutive 25 m lines, separated by approximately 5 m, were deployed along depth contours. Coral colony density was documented along the two 25 m belt transects lines by enumerating all coral colonies whose center fell within 0.5 or 1 meter on either side of the transect line. Coral cover was documented by point-intercept method at 50 cm intervals along both 25-m lines. Disease assessments were conducted along the two belt transects but within a wider 6 meter area (25×6 m). Disease prevalence is relatively low in Hawaii and it was felt that a larger search area (wider belt) was necessary to get a more accurate account of disease presence. Belt length and width were modified as needed to accommodate time constraints underwater and so varied among sites. All coral colonies with lesions were enumerated and photographed, and samples were collected for follow-up histopathological analyses. Lesions were classified as tissue loss, discoloration or growth anomalies and described as per Work and Aeby [38]. These protocols have been used in numerous other studies to document coral cover, community structure and disease within the NWHI [39], [40]. From these surveys we documented reef characteristics (depth, coral cover, colony density) as well as differences in disease distribution and prevalence across the archipelago.

**Table 1:** Disease surveys conducted in the main and northwestern Hawaiian Islands in 2004 and 2005.

MHI	# sites surveyed	depth range (m)	total area surveyed for disease (m^2^)
Hawaii	19	7.3–15.2	4,978
Maui	11	2.1–15.2	3,150
Oahu	27	1.5–18.3	7,872
Kauai	12	6.4–17.1	3,600
Niihau	6	9.1–15.2	1800
Lehua	3	11.6–15.2	900
**total**	**78**		**22,300**
**NWHI**			
French Frigate Shoals	11	1.5–11.3	3,000
Gardner Pinnacles	3	12.2–19.5	900
Maro Reef	8	10.7–18.3	1650
Laysan	3	12.2–14.6	750
Lisianski	9	9.1–17.1	1800
Pearl & Hermes	13	0.9–15.8	3600
Midway	9	0.9–14.3	2550
Kure	8	1.5–14.9	2175
**total**	**64**		**16,425**

### Geospatial Environmental Predictors

Geospatial environmental predictors included frequency of weekly sea surface temperature anomalies (WSSTA) and frequency of erythemal surface ultraviolet (UV) radiation anomalies, while human population size served as a proxy for the impact of anthropogenic effects. Coral disease survey locations were imported as geo-referenced points into the GIS and predictor values were extracted for each survey. Human population counts were raster data of 2.5 arc-minutes resolution adjusted to match UN totals for 2005 [41]. Human population size was summed within circular buffers of 1 and 100 km around each survey site. Data were included for all grid cells that intersected a buffer. The mean annual WSSTA values for the four years prior to the year of the survey were extracted for each coral survey location. The frequency of weekly sea surface temperature anomalies (WSSTA) was defined as the number of times over the previous 52 weeks that the weekly sea surface temperature (SST) minus the weekly climatological SST, equaled or exceeded 1uC [42]. SSTA data were approximately 4 km resolution Pathfinder AVHRR raster data on a weekly time scale from 1985 through 2005. The frequency of erythemal surface ultraviolet (UV) radiation anomalies were the number of times between 2000 and 2004 that the monthly average exceeded the climatological mean plus one standard deviation [43]. These values were summed across the 12 months to provide a single value, ranging from 0–19, representing the number of anomalous values for each coral survey location over the entire 5 years. The erythemal surface UV data were measured as part of the GSFC TOMS EP/TOMS satellite program at NASA [44]. These data were processed by NASA to isolate the amount of erythemal ultraviolet (UV) light that reaches Earth's surface. Data were reported as the average Joules (J) per m^2^ per month at one-degree cell (110 km by 110 km) resolution. All data were prepared and geoprocessed with ArcGIS 9.2 and Matlab 7.1.

### Statistical analyses

Time constraints underwater prevented the enumeration of all coral colonies within the wider belt transects surveyed for disease. Therefore, we estimated the total number of colonies surveyed for disease based upon the mean number of colonies m^−2^ found within the narrower (25×0.5 m or 25×1 m) belt transects. Prevalence of disease was defined as the proportion of colonies surveyed that had a particular lesion type. Overall prevalence was the proportion of colonies surveyed that had a particular lesion type with all surveys combined. Frequency of disease occurrence (FOC) was calculated as the percent of sites surveyed having one or more coral colonies manifesting a particular disease state. Disease susceptibility can vary between coral genera so a Chi-square test for equality of distributions was used to examine potential differences between observed prevalence of a disease and expected prevalence based on the abundance of the affected coral genera (each region was examined separately). All calculations for disease prevalence or FOC were done by coral genera (e.g. prevalence of *Porites* trematodiasis = (# infected *Porites* colonies/total # *Porites* colonies surveyed) * 100).

#### Examining disease assemblage patterns (multivariate analyses)

Differences in disease assemblages were investigated using multivariate community analyses. These types of analyses have been a foundation of ecological investigations for many years, although few studies have used them to investigate coral disease patterns (but see [13], [30]). Initially, we examined differences in disease assemblages (using raw counts of diseased colonies standardized by survey area as the multivariate response) at two factor levels, Region (NWHI *versus* MHI) and Island (14 islands within the archipelago). Prior to the multivariate analyses, the raw count data were subjected to a dispersion-weighting pre-treatment [45] (analyzed separately for each factor level) to account for any significant levels of spatial clustering for each disease between replicate sub-samples within each *a priori* defined factor (i.e. between islands when analyzing at the Region level, and between transects when analyzing at the Island level). Tests were based on 1000 permutations of the raw data. *Porites* trematodiasis showed the highest levels of spatial clustering. We then used a permutational multivariate analysis of variance (PERMANOVA) [46], [47] to test for differences between the two factors, with Island nested within Region. At this stage we did not create factors for either season or year as these were tested as categorical predictors during the modeling stages of the analyses (see below). The PERMANOVA analysis was based on a zero-adjusted Bray-Curtis similarity matrix [48], type III partial sums of squares, and 9999 random permutations of the residuals under the reduced model. PERMANOVA pair-wise interactions were used to identify significant differences in disease assemblages between islands within each region.

To identify indicator diseases between the two regions (those contributing most to the patterns in multivariate space), we used a constrained canonical analysis of principal coordinates (CAP) [49], [50]. We calculated Spearman rank correlations of the canonical ordination axes with the original disease variables. Diseases with strong correlations (defined as ≥0.7 in this study) were then overlaid as a bi-plot. A similarity percentages (SIMPER) analysis [51] was used to identify indicator diseases between islands within each region. SIMPER is not a statistical test, but is useful for initial data exploration.

#### Modeling disease-environment associations

Three regional indicator diseases were identified in the multivariate analyses, which were then modeled against 14 predictor variables (Table 2). Predictor variables included biotic and abiotic variables that could affect disease processes or help explain presence of disease and included coral host abundance, measures of overall coral reef state (coral cover, macroalga cover), potential stressors (weekly sea surface temperature anomalies (WSSTA)), ultraviolet radiation input, local (within 1 km of the site) and regional (within 100 km) human population sizes and variables involved with sampling design, such as month or year of surveys and survey effort. To investigate the association of the abundance of each disease with the predictor variables, we used a permutational distance-based multiple regression technique (DISTLM) [47]. The technique is robust to zero-inflated data sets, such as ours, and makes no assumptions about the distribution of the response variable (normality does not have to be satisfied). No two predictors exceeded an inter-correlation value of 0.75. Predictors were normalized and fitted conditionally in a step-wise manner, with tests based on 9999 permutations of the residuals under the reduced model [47]. Model selection was based on Akaike's Information Criterion [52] with a second-order bias correction applied (AICc) [53], [54]. To interpret the relationship between disease prevalence and the optimal predictor(s), we used distance-based redundancy analysis plots (dbRDA) [47]. We modeled two response variables for each disease: prevalence (proportion of hosts found to be diseased) and diseased colony raw counts. This was to examine for differences between the two model responses but also to allow disease abundance to be standardized against area surveyed (survey effort) in the case of the raw counts. Modeling analyses were based on zero-adjusted Bray-Curtis similarity matrices [48] and conducted in PRIMER v6 [51] and PERMANOVA+ [55]. All models, raw counts/prevalence, were created for each coral disease separately, as recommended by Williams et al. [32].

**Table 2:** Predictor variables used in the modeling analyses with their codes and units.

Variable	Code	Description and units	Min	Max
*Porites* cover	PorCov	% cover	< 1	91
*Porites* density	PorDen	# colonies/m^2^	< 1	10.4
Overall coral cover	OverallCov	% cover	< 1	91
Overall coral density	OverallDen	# colonies/m^2^	< 1	21
Macroalgae cover	Algae	% cover		
Depth	Depth	m	1	18
WSSTA frequency for survey year	WSSTAyr	number of events	0	22
WSSTA frequency during prior 4 years prior to survey year	WSSTAfour	mean number	2	19
Human numbers within 1 km	HumPop1	number of people	0	32,406
Human numbers within 100 km	HumPop100	number of people	0	954,480
UV input	UV	rating scale	0	10
Month	Month	month of survey	–	–
Year	Year	year of survey	–	–
Survey effort	Area	m^2^ of reef	150	300

Min/Max, minimum and maximum predictor values between sites.

## Results

### Lesion descriptions and overall disease occurrence across the Hawaiian archipelago

**Figure 2:**
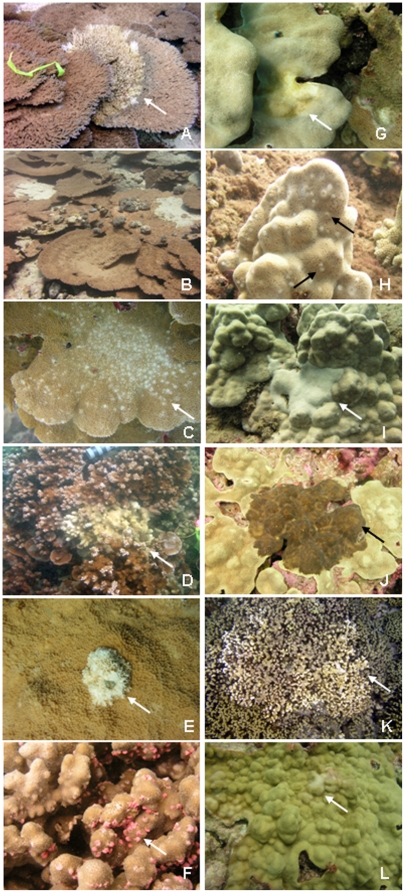
Photos and description of coral diseases observed during surveys across the Hawaiian archipelago. All lesions have been characterized histologically and those results will be presented elsewhere. A) *Acropora* white syndrome (AcroWS): diffuse areas of acute to subacute tissue loss, B) *Acropora* growth anomalies (AcroGA): protuberant growths of skeleton accompanied by aberrant calyx formation overlaid by normally pigmented to colorless tissues, *C) Montipora* multifocal tissue loss (MontMFTL): multiple (>5) variably sized areas of acute to subacute tissue loss, D) *Montipora* white syndrome (MontWS): one to 5 areas of acute to subacute tissue loss, E) *Montipora* growth anomalies (MontGA): protuberant growths of skeleton accompanied by aberrant calyx formation overlaid by normally pigmented to colorless tissues, F) *Porites* trematodiasis (PorTrem): multiple small (∼5 mm) swollen pink to white nodules, G) *Porites* tissue loss syndrome (PorTLS): one to 5 areas of acute to subacute tissue loss, H) *Porites* multi-focal tissue loss (PorMFTL): multiple (>5) variably sized areas of acute to subacute tissue loss, I) *Porites* growth anomalies (PorGA): protuberant growths of skeleton accompanied by aberrant calyx formation overlaid by normally pigmented to colorless tissues, J) *Porites* brown necrotizing disease (PorBND): diffuse areas of unidentified brown homogenous matrix (not algae) obliterating underlying area of tissue loss and well delineated from surrounding normal tissue, K) *Porites* bleaching with tissue loss (Por bl w/TL): focal areas of bleaching with diffuse areas of acute to subacute tissue loss, L) *Porites* discolored tissue thinning syndrome (PorDTTS): distinct areas of tissue thinning and pallor. Arrows indicate lesions.

Twelve types of lesions were identified from the 3 coral genera, *Porites, Montipora,* and *Acropora,* from across the archipelago (Fig. 2). Eight diseases were documented from reefs within the MHI and 10 diseases from the NWHI with overlap in types of diseases (6 of 12) between regions. Signs of coral disease were widespread occurring at 87.2% of the sites surveyed within the MHI and 80% within the NWHI. Frequency of occurrence varied between diseases with some diseases, such as *Porites* trematodiasis (PorTrm), found archipelago-wide whereas other diseases, such as growth anomalies, found predominantly within one region (MHI) (Table 3). Average prevalence of all diseases, except PorTrm, was low (<1%) (Table 4). The average prevalence of PorTrm across the archipelago was 5.3% (range 0–87%).

**Table 3:** Frequency of occurrence (FOC) of different coral diseases within the main and northwestern Hawaiian Islands.

	MHI	NWHI
Por Trem	67.5	75.4
Por GA	59.7	4.9
Por TLS	22.1	32.8
Por bl TL	9.1	0
Por MFTL	6.5	0
Por DTTS	0	19.7
Por BND	0	3.3
Mont GA	26.9	4.5
Mont WS	14.1	27.3
Mont MFTL	14.1	11.4
Acro WS	-	33.3
Acro GA	-	22.2

FOC represents the proportion of total surveys (%), which contained corals (presence/absence) affected by each particular disease. 78 sites were surveyed within the main Hawaiian Islands and 64 sites surveyed in the northwestern Hawaiian Islands.

Por = *Porites*, Mont = *Montipora*, Acro = *Acropora*, Trem = trematodiasis, GA = growth anomaly, TLS = tissue loss syndrome, MFTL = multi-focal tissue loss, bl = bleaching, DTTS = discolored tissue thinning syndrome, BND = brown necrotizing disease, WS = white syndrome. ‘-‘ indicates that particular coral genera was not present in transects.

**Table 4:** Average prevalence of diseases found on the reefs within the main and northwestern Hawaiian Islands.

	MHI	NWHI
Por Trem*	1.1 (0.3)	10.7 (2.2)
Por GA *	0.64 (0.15)	0.32 (0.3)
Por TLS	0.11 (0.03)	0.82 (0.28)
Por bl TL	0.04 (0.03)	0
Por MFTL	0.006 (0.003)	0
Por DTTS	0	0.31 (0.12)
Por BND	0	0.02 (0.02)
Mont GA	0.06 (0.02)	0.009 (0.009)
Mont WS	0.06 (0.02)	0.17 (0.06)
Mont MFTL	0.0007 (0.0007)	0.35 (0.2)
Acro WS	-	0.76 (0.4)
Acro GA	-	0

78 sites were surveyed within the main Hawaiian Islands and 64 sites surveyed in the northwestern Hawaiian Islands. Standard error in parentheses. * indicates a significant difference between regions based upon the Wilcoxon two sample test. Por = *Porites*, Mont = *Montipora*, Acro = *Acropora*, Trm = trematodiasis, GA = growth anomaly, TLS = tissue loss syndrome, MFTL = multi-focal tissue loss, bl = bleaching, DTTS = discolored tissue thinning syndrome, BND = brown necrotizing disease, WS = white syndrome. ‘-‘ indicates that particular coral genera was not present in transects.

### Differences in disease among coral taxa

**Figure 3:**
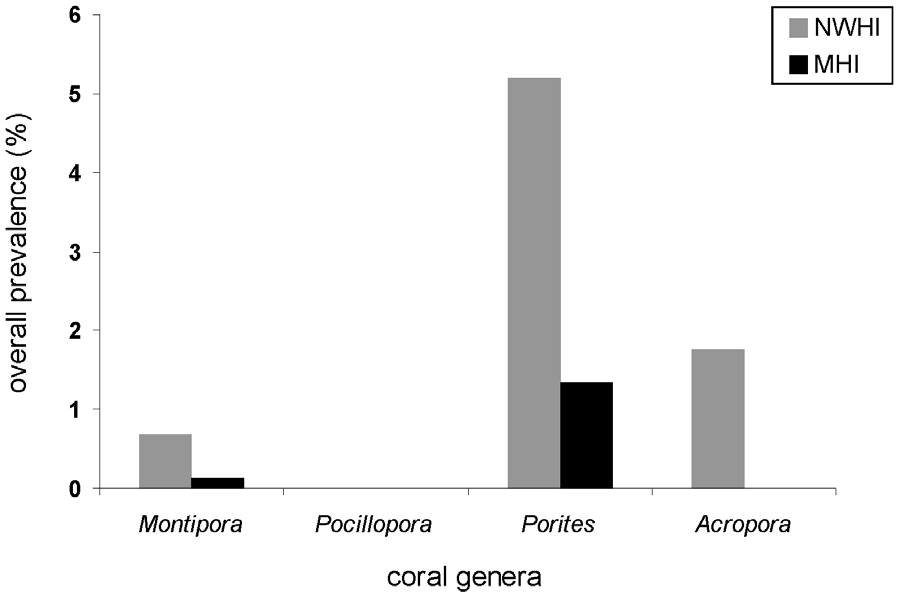
Differences among coral taxa in disease prevalence across the Hawaiian archipelago.

Coral taxa differed in manifestation of lesions with seven diseases described from *Porites* (PorTrem, PorGA, PorMFTL, PorTL, Por bl w/TL, Por BND, Por DTTD), three from *Montipora* (MontWS, MontMFTL, MontGA) and two from *Acropora* (AcroWS, AcroGA) (Fig. 2). No disease signs were found on *Pocillopora* during these surveys. Prevalence of disease also varied among coral taxa with *Porites* having the highest prevalence and *Pocillopora* the lowest (Fig. 3) and these differences were consistent across regions (MHI: *X* ^2^ = 1184.8, df = 2, p<0.001; NWHI: *X* ^2^ = 928.4, df = 3, p<0.001).

### Disease assemblage patterns between regions

**Figure 4:**
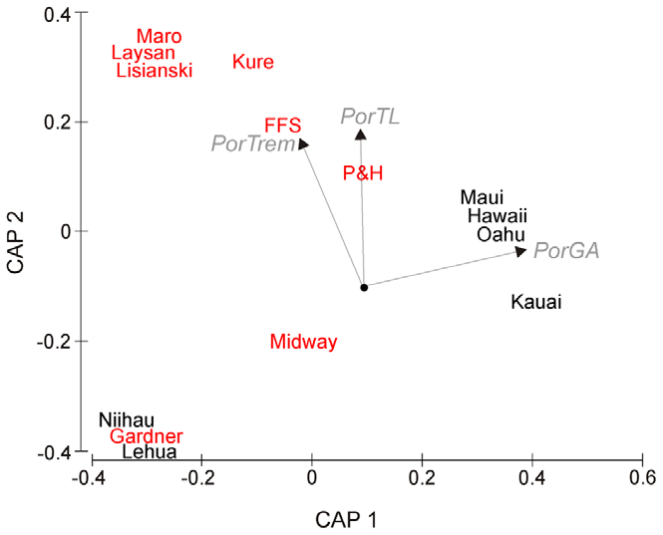
Constrained CAP ordination of coral disease assemblages for 136 sites at 14 islands across the Hawaiian archipelago. Group centroids are displayed for each island (MHI – black, NWHI – red). Ordination is based on a zero-adjusted Bray-Curtis coefficient. Bi-plot indicates the disease variables (vectors) exerting the strongest influence on the patterns in multivariate space (in grey). The length and direction of each vector indicates the strength and sign, respectively, of the relationship between that disease variable and the CAP axes. Note that *Acropora* diseases are not included in the analysis. FFS, French Frigate Shoals; P&H, Pearl and Hermes. PorTrem, *Porites* trematodiasis; PorTL, *Porites* tissue loss; PorGA, *Porites* growth anomalies.

Disease assemblages differed significantly between regions (*Pseudo-F* = 9.905, P = 0.0001), with three diseases, *Porites* trematodiasis (PorTrem), *Porites* growth anomalies (PorGA), and *Porites* tissue loss syndrome (PorTL) contributing most strongly to driving this separation (Fig. 4). Increased levels of PorTrem were associated with the Northwestern Hawaiian Islands (French Frigate Shoals, Kure, Maro, Laysan and Lisianski) and average prevalence of PorTrem was significantly higher in the NWHI (10.7±2.2%) compared to the MHI (1.1±0.3%) (Wilcoxon two sample test, W = 4756, p<0.001; Table 4). PorGAs were positively associated with the main Hawaiian Islands (Maui, Hawaii, Oahu, and Kauai) and the average prevalence of PorGAs was significantly higher in the MHI (0.64±0.15%) as compared to the NWHI (0.32±0.3%) (Wilcoxon two sample test, W = 3177, p<0.001; Table 4). The patterns of PorTLS prevalence were more difficult to interpret but seemed to be positively associated with some islands within the Northwestern Hawaiian Islands (Pearl and Hermes, and to a lesser extent French Frigate Shoals, Kure, Lisianski, Laysan and Maro), but negatively associated with other islands (Midway and Gardner) and the islands of Niihau and Lehua within the MHI (Fig. 4). Average prevalence of PorTLS did not differ between regions (Wilcoxon two sample test, W = 4990, p = 0.08; Table 4).

### Disease assemblage patterns within regions

Disease assemblages differed significantly between islands within each region (*Pseudo-F* = 3.208, P = 0.0001; Table 5). Within the main Hawaiian Islands, differences in disease assemblages between islands were predominantly driven by PorGAs, with the disease particularly characteristic of Oahu and Maui (Table 6 & Fig. S1). PorTrem was also an influential disease, particularly characteristic of Hawaii (Table 6 & Fig. S1). Within the northwestern Hawaiian Islands, PorTrem was consistently an important indicator disease separating islands (Table 6 & Fig. S2). The disease was particularly characteristic of Laysan and Maro. *Montipora* white syndrome (MontWS) was also an important indicator disease, separating Maro out from the other islands (Table 6 & Fig. S2).

**Table 5:** Average similarity (%) in coral disease assemblages among islands within (a) the Main Hawaiian Islands and (b) the Northwestern Hawaiian Islands.

(a)	Maui	32.0						
	Oahu	32.0	32.5					
	Niihau	**32.5** *	**19.4** *	**28.5** *				
	Kauai	37.6	**31.9** *	35.3	**48.9** *			
	Lehua	34.3	**21.8** *	**30.1** *	80.6	50.3		
		Hawaii	Maui	Oahu	Niihau	Kauai		
(b)	Gardner	44.1						
	Maro	**24.8** *	**7.6** *					
	Laysan	24.7	10.9	52.4				
	Lisianski	**27.9** *	**12.0** *	56.4	48.7			
	P&H	36.6	43.4	**29.2** *	26.1	**28.5** *		
	Midway	43.4	63.7	**11.3** *	**15.6** *	**16.8** *	41.4	
	Kure	30.0	34.7	**37.2** *	32.7	32.4	32.1	**31.6** *
		FFS	Gardner	Maro	Laysan	Lisianski	P&H	Midway

*indicates significantly different from each other (P≤0.01) using permutational analysis of variance pairwise comparisons. Analyses based on 9999 random permutations. P&H, Pearl and Hermes.

**Table 6:** Indicator diseases important in explaining differences in disease assemblages among islands within the main and northwestern Hawaiian Islands.

Region	Significant pairwise interaction	Indicator diseases (in order of importance)	Characteristic of
**MHI**	Niihau - Hawaii	PorTrem, PorGA, MontGA, PorMFTL	Hawaii
	Niihau - Maui	PorGA, MontGA, PorTrem, MWS	Maui
	Niihau - Oahu	PorGA, PorTrem, MWS, PorTLS	Oahu
	Kauai - Maui	PorGA, MontGA, PorTrem, MWS	Maui
	Kauai - Niihau	PorTrem, PorGA	Kauai
	Lehua - Maui	PorGA, MontGA, PorTrem, MWS	Maui
	Lehua - Oahu	PorGA, PorTrem, MWS, PorTLS	Oahu
**NWHI**	Maro - French Frigate Shoals	PorTrem, MWS	Maro
	Maro - Gardner	PorTrem, MWS	Maro
	Lisianski - French Frigate Shoals	PorTrem	Lisianski
	Lisianski - Gardner	PorTrem	Lisianski
	Pearl & Hermes - Maro	PorTrem, MWS	Maro
	Pearl & Hermes - Lisianski	PorTrem	Lisianski
	Midway - Maro	PorTrem, MWS	Maro
	Midway - Laysan	PorTrem	Laysan
	Midway - Lisianski	PorTrem	Lisianski
	Kure - Maro	PorTrem, MWS	Maro
	Kure - Midway	PorTrem	Kure

### Disease-environment associations

#### *Porites* trematodiasis

PorTrem prevalence was optimally predicted by survey area (explaining 19.4% of the variation in prevalence), with prevalence decreasing as survey area increased (Table 7). However, when the number of cases of PorTrem, were standardized against survey area, *Porites* cover was the optimal predictor (explaining 15.9% of variation in prevalence); with cases of PorTrem positively associated with increases in *Porites* cover. PorTrem prevalence also increased as human population numbers within a 100 km radius decreased and where *Porites* colony densities were reduced (i.e. relatively few, but large colonies present) (Table 7).

**Table 7:** Summary results of a distance-based permutational multiple regression analysis for the association of three coral diseases with 14 predictor variables across 134 sites throughout the Hawaiian archipelago.

Response	Disease	Predictor	AICc	Pseudo-F	P value	% variability	% total	Relationship
Prevalence	PorTrem	Area	959.90	32.057	0.0001	19.4		negative
		PorCov	951.11	11.088	0.0002	6.2		positive
		HumPop100	937.44	5.5827	0.0002	5.4		negative
		PorDen	935.66	2.228	0.0043	2.8	33.8	negative
Counts	PorTrem	*Porites* cover	761.40	25.163	0.0001	15.9		positive
		WSSTA 4 yr	750.11	13.754	0.0001	7.9		unclear
		WSSTA	742.03	10.283	0.0010	5.5	29.3	negative
Prevalence	PorGA	WSSTA 4 yr	750.68	23.17	0.0001	14.9		negative
		Depth	745.11	10.775	0.0005	5.7		negative
		UV	728.91	7.7012	0.0038	4.7		unclear
		OverallCov	739.68	7.5454	0.0042	4.4		positive
		WSSTAyr	737.55	4.1955	0.0310	2.4		negative
		HumPop100	722.75	4.6514	0.0229	2.3	34.4	positive
Counts	PorGA	WSSTA 4 yr	480.93	26.301	0.0001	16.6		negative
		UV	470.99	12.305	0.0006	7.2		positive
		Overall coral cover	464.43	8.7022	0.0035	4.8		positive
		Depth	452.5	9.7621	0.0012	4.8		negative
		WSSTAyr	460.15	6.349	0.0113	3.4		unclear
		Humans 100 km	445.5	5.3499	0.0210	2.5	39.3	positive
Prevalence	PorTL	Area	754.58	25.344	0.0001	16.1	16.1	negative
Counts	PorTL	Overall coral cover	379.28	6.6851	0.0107	4.5		positive
		WSSTAyr	376.68	4.6629	0.0311	3.1	7.6	negative

Two response variables are modeled for each disease; prevalence and the number of disease cases. The optimal predictors of each response and the proportion of variability in the data set they explained (% variability) are shown. Where possible, the relationship between the response and predictor is displayed. Model development was based on step-wise selection and Akaike's Information Criterion with a second-order bias correction applied (AICc), with the total variation explained (r^2^) by each model shown (% total). Analyses based on 9999 permutations of the residuals under a reduced model.

#### *Porites* growth anomalies

PorGA abundance (both prevalence and number of cases) was optimally predicted by the average number of WSSTAs in the four years prior to year of surveying, explaining 14.9% and 16.6% of the variation in the two model responses, respectively (Table 7). PorGA abundance decreased as the mean frequency of WSSTAs increased. PorGA abundance was also higher in shallower depths and was positively associated with overall coral cover, frequency of UV radiation anomalies and human population sizes within a 100 km radius (Table 7).

#### *Porites* tissue loss syndrome

PorTLS prevalence was optimally predicted by survey area (explaining 16.1% of variation in prevalence), and prevalence decreased as survey area increased (Table 7). When standardized for differences in survey area, the number of cases of PorTLS was positively associated with overall coral cover and negatively associated with the number of WSSTAs during the year of survey, although the predictors explained little of the overall variation (Table 7).

### Model performance

Model performance varied among diseases. When averaging across models for both disease prevalence and number of cases, PorGA was most effectively modeled (36.9% overall variation explained) and PorTLS least effectively modeled (11.9%). Across all three diseases and averaging across the two model responses, mean overall explained variability in disease abundance was 26.7% with unexplained variability equaled to 73.3%.

## Discussion

We surveyed 142 sites from across the Hawaiian archipelago, which spanned a wide range of biotic and abiotic conditions. Twelve different coral diseases were found in three coral genera (*Porites, Montipora, Acropora*). Reports of diseases with similar field signs have been reported from numerous regions across the Indo-Pacific and include the tissue loss diseases (PorTLS, Por bl w/TL, PorMFTL, MontWS, MontMFTL and AcropWS) and the growth anomalies (PorGA, MontGA, AcropGA) [10], [11], [13], [16], [18], [29], [30], [56], [57]. Unfortunately, regional comparisons of coral disease are difficult due to differences in nomenclature of coral diseases and the paucity of information on ecology and etiology of coral diseases [23], [38]. For example, several studies report all tissue loss diseases as “white syndrome” regardless of whether the pattern of tissue loss is diffuse, focal, or multi-focal [10], [13], [18], [29], [30]. The other difference in manner of reporting diseases is indicating whether or not field signs suggest transmissibility between coral genera (i.e. whether or not there is evidence of disease progression between two adjacent coral colonies from different coral genera). In some field studies, disease can be found progressing from one colony to others regardless of coral genera and so those could be considered non-host specific “white syndromes” (Dalton and Smith 2006, pers. obs.). In contrast, in Hawaii diseased colonies have been observed adjacent and touching healthy colonies of a different genus with no disease transmission ever apparent. Given these field signs, it is be more informative to include the host genus in the disease name (e.g. *Acropora* white syndrome) [38].

Surveys also only offer a snapshot in the progression of diseases and so limit accurate disease diagnosis. For example, we found *Porites* multi-focal tissue loss, which has disease signs similar to *Porites* ulcerative white spot disease (PUWS) described from reefs in the Philippines [58]. However, we did not follow disease progression to determine whether it was consistent with PUWS or conduct follow-up laboratory analyses, which would be required to properly identify PUWS.

In general, the average prevalence of diseases on reefs within the Indo-Pacific are low [10], [11], [13], [17], [18], [19], [40], which is consistent with our findings for disease, excluding PorTrm, on corals within the Hawaiian archipelago. However, some coral diseases, such as PorTrm, can be quite prevalent in the Indo-Pacific at the local level. We found PorTrm, the most common and prevalent disease in Hawaii, averaged 5.3% (range  = 0 to 87%). Within the Philippines, Kaczmarsky [12] reported PUWS in up to 53.7% of the *Porites* colonies surveyed. In Guam, Myers and Raymundo [29] reported the average prevalence of white syndrome to be 8.9% (range  = 0–26.3%) and PUWS averaged 2.8% prevalence (range  = 0–17.4%).

Similar to several studies across the Indo-Pacific differential disease susceptibilities were found among coral taxa. *Porites* is the dominant coral on the reefs of the Hawaiian archipelago comprising 63.5% of the overall coral community within transects (data not shown). Accordingly, in both regions *Porites* had the highest number, widest distribution, and highest prevalence of diseases as compared to other coral taxa. Kaczmarsky [12] found high levels of disease in *Porites* in the Philippines where *Porites* is also the dominant coral. *Acropora,* although a small component of the Hawaiian coral reef community, had the second highest prevalence of disease among coral taxa. This is consistent with other studies, which have found *Acropora* to be especially vulnerable to disease in many regions of the Indo-Pacific [10], [13], [40], [59] as well as the Western Atlantic [7], [8], [60], [61]. In contrast, no signs of disease were found in *Pocillopora* in either region, MHI or NWHI. The Australian GBR is a region where pocilloporids are a small component of the coral community, yet have high disease prevalence [10]. Interestingly, skeletal eroding band is the most prevalent disease on *Pocillopora* on the GBR and while this disease was reported once from Hawaii based on presence of ciliates on bare coral skeleton [62] it has not been verified using observational data (evidence of disease progression) or histological techniques.

The effect of environmental stress on the dynamics of infectious disease can vary depending on the underlying host-pathogen-stress dynamics [22]. For example, if an environmental stress reduces host abundance below a threshold level then disease prevalence may decline. Conversely, if the stress compromises the host increasing its susceptibility to disease, then prevalence might increase. Accordingly, we found distinct regional differences when the disease assemblage patterns were examined across the archipelago. PorGAs were significantly more prevalent within and indicative of the human-impacted reefs of the MHI but PorTrm was reduced within the MHI. Instead, PorTrm was significantly more prevalent within and indicative of the pristine reefs of the NWHI. PorTLS was also found to be important in driving the regional differences, although that relationship was less clear. These results, although surprising and unexpected, highlight the importance of understanding disease ecology when interpreting patterns of disease occurrence.

PorTrm is caused by the digenetic trematode, *Podocotyloides stenometra* Pritchard [63], [64], [65]. *P. stenometra* has a complex life cycle involving a first intermediate host thought to be a mollusk, *Porites* as the second intermediate host, and coral-feeding fish as the final host [64]. Completion of the parasite's life cycle occurs when coral-feeding fish ingest the infected polyp, with the adult worm subsequently residing in the guts of fish [64]. Multiple species of coral-feeding reef fish can serve as host to the parasite [64], [66]. As such, occurrence of this disease on the reefs requires the presence of all three of its hosts (mollusk, coral and fish) and so presumably, a healthier ecosystem supporting a greater diversity of vertebrate and invertebrate hosts would, in turn, support a greater abundance of their parasites [67]. Using statistical modeling, we found PorTrm, when survey area was controlled for, showed strong positive associations with host abundance (% cover), consistent with other studies [32], [68] and a negative association with human population size. The NWHI is a healthier ecosystem than the MHI [69] and has a higher abundance and diversity of reef fish. Friedlander and DeMartini [36] found the mean fish standing stock to be 260% higher in the NWHI as compared to the MHI, and butterflyfish are thought to be the main vector transmitting PorTrm across the reefs [68]. We suggest that environmental conditions within the MHI, which also affect host abundances, are not conducive to widespread transmission of this parasite, a phenomenon similar to that noted by others where environmental degradation is associated with reduced parasite diversity [67]. The pattern of increased PorTrm in healthier ecosystems was also documented in Kaneohe Bay where southern reefs, heavily impacted by runoff [70], had significantly lower levels of PorTrm than relatively cleaner northern reefs [68]. Whereas for many coral diseases, stressors clearly play a role in increasing disease levels, this does not hold true for PorTrm. In fact, a high prevalence of PorTrm on Hawaiian reefs appears to be indicative of a comparatively healthier coral reef ecosystem. Fortunately, PorTrm is a chronic disease that, although it reduces coral growth [71], [72], does not usually result in colony mortality. Interestingly, the model also showed a negative association between PorTrm prevalence and size of area surveyed (e.g. the larger the area surveyed the lower the overall prevalence of PorTrm) and this is probably due to the spatially clumped nature of PorTrm infections [68].

In contrast, PorGAs were strikingly more common and prevalent within the MHI suggesting that this disease could be affected by environmental conditions associated with human presence. Kaczmarsky [12] reported a high prevalence of PorGAs (up to 39.1%) in the Philippines, a region that contains some of the most impacted reefs in the world [73]. The etiology of GAs is unknown, however Kaczmarsky and Richardson [74], through controlled experiments, found that growth anomalies in *Porites* might be transmissible. Little else is known about the ecology of PorGAs making interpretation of these patterns challenging. However, we found PorGAs prevalence showed positive associations with overall coral cover, the frequency of UV anomalies and human population size. Aeby et al. [75] examined PorGAs from 13 regions across the Indo-Pacific and found that prevalence of PorGAs was strongly host density-dependent and also showed strong positive associations with human population size. Our study adds to the growing body of evidence that suggests that PorGAs are related, directly or indirectly, to some environmental co-factor associated with increased human population size at local and regional spatial scales. The underlying mechanism influencing the association between PorGAs and human density is unclear and warrants further investigation.

Other hypotheses have been proposed to explain the distribution of coral growth anomalies. Damage to cells from ultraviolet radiation was suggested as a potential mechanism contributing to formation of GAs in *Acropora* [76]. However, Stimson [77] found no effect of UV exposure on the development of GAs on *Porites compressa* in Hawaii. We found a negative association between PorGAs and depth, which is consistent with other studies [32], [78], and a positive association with UV. However, other environmental variables can also vary with depth or in areas with higher UV exposure. For example, corals on a shallow reef as compared to a deeper reef usually experience higher water motion, UV exposure, ambient light and greater temperature fluctuations. The ecological mechanisms behind these disease-environment patterns are likely to be complex and could be the result of direct or indirect associations. A better understanding of disease etiology should help in the interpretation of these patterns and should be the focus of future studies. Regardless of the underlying drivers, the high prevalence of PorGAs within the MHI is a concern. Growth anomalies have been linked to reduced growth in affected corals [79], a reduction in the amount of lipids [80], reduced reproduction [57], [80] and an impaired ability to withstand bleaching stress [81]. As such, more research is needed to understand the effects of growth anomalies on coral populations in Hawaii. Understanding disease processes becomes especially important in light of predictions of increased severity and frequency of coral bleaching associated with global climate change [28] and continual increases in local stressors on the reefs of Hawaii.

PorGAs and PorTrem were also found to be important in explaining differences among disease assemblages between islands within regions. Within the MHI, Oahu and Maui stand out as the most affected by disease with PorGAs especially prevalent. Not surprisingly, Oahu and Maui also contain the highest human population densities (567/km^2^ and 62/km^2^, respectively) as compared to the other islands (Kauai: 41/km^2^, Hawaii: 14/km^2^, Niihau: 1/km^2^, Lehua: 0/km^2^) (http://geonames.usgs.gov). PorTrm was more characteristic of Kauai and Hawaii, which have lower human densities and host abundance adequate to maintain disease in the population. Niihau and Lehua have few human influences but also low (<1%) coral cover (host abundance) and accordingly low disease prevalence. Within the NWHI where human influences are minimal, PorTrem was the main disease underlying differences in disease assemblages among islands and was especially prevalent on Maro, Lisianski, Laysan and Kure. This study, as well as others, have found host abundance to be an important factor affecting PorTrem prevalence and this may help explain disease differences among islands as Maro and Lisianski have the highest coral cover among islands (avg. 40.7% and 38.3%, respectively) (Fig. S3). However, neither Laysan (avg. 16.4%) nor Kure (avg. 12.8%) had high abundance of coral cover, suggesting other factors not considered in this model may also be important. Coral-feeding reef fish have been suggested as a vector for PorTrem [68] and therefore abundance of coral-feeding reef fish could also affect disease prevalence and should considered in future studies.

Maro and Midway had the highest prevalence of *Montipora* white syndrome (MontWS) within the archipelago. MontWS was found to be host density dependent in Kaneohe Bay, Oahu [31] and the sites we surveyed at Maro and Midway contained some of the highest cover of *Montipora* within the NWHI (Fig. S3). However, Williams et al. [32] also found MontWS was positively associated with chlorophyll-*a* concentrations, which would be indicative of poor water quality. Water clarity is reduced at Maro as the structure of Maro is complex (combination of linear and patch reefs) which allows wave energy to penetrate lagoonal water, which keeps fine sediments suspended in the water column [39], another stressor that may explain the higher occurrence of MontWS on Maro's reefs. At Midway, montiporids are found almost exclusively in the backreefs [82], which suffered severe bleaching in 2002 [83] and again in 2004 [84]. For MontWS, host abundance and coral stress may both be contributing to disease prevalence.

Coral diseases, like most diseases, display complex association with their environment because of the intricate nature of the host-environment-agent triad and the inherent multi-collinearity present between biotic and abiotic variables in any ecological system [20], [32], [38], [85]. Not surprisingly, using statistical modeling we found similarities and differences among the three indicator diseases with respect to potential underlying drivers. All three diseases showed positive associations with measures of host abundance (*Porites* cover and overall coral cover) which is consistent with infectious disease ecology theory [85]. Many examples of relationships between host abundance and disease prevalence exist throughout a wide range of ecosystems including coral disease [28], [29], [31], [32], [75]. All three diseases were also consistent in showing a negative association with WSSTA, which contrasts with other coral disease studies that have found a positive association between coral disease and thermal stress [28], [86], [87], [88]. It may be that chronic diseases, such as GAs or Trematodiasis are less influenced by temperature when compared to the tissue loss diseases, many of which are caused by pathogenic bacteria with virulence factors that may be enhanced at higher temperatures [8], [14], [89], [90]. Aeby et al. [75] also found no association between WSSTAs and prevalence of *Acropora* and *Porites* growth anomalies across the Indo-Pacific. However, we also found a negative association between WSSTAs and PorTLS. Nothing is known about the etiology or pathogenesis of PorTLS making interpretation of these associations difficult but not all tissue loss diseases necessarily respond to temperature stress. Aeby et al. [31] found no evidence of seasonality in the tissue loss disease, MontWS, in Hawaii. However, they hypothesized that evidence of seasonality could have been obscured by different stresses in each respective season (e.g. temperature stress in summer months and heavy rainfall and the associated stress of terrestrial run-off in winter months). Differences in underlying associations among the three indicator diseases also occurred with human population size, with a positive association with PorGA and a negative association with PorTrm but no significant explanatory power for PorTLS. Clearly disease processes are complex and our results are consistent with the idea that different coral diseases can show distinct associations with multiple environmental factors [32], [75]. We also found that the overall unexplained variability in disease abundance among the three indicator diseases was high (a mean of 73.3% across all three diseases) suggesting that much more research is needed in understanding the pathogenesis of these diseases.

Regardless of the underlying drivers, this study did find strong patterns of disease occurrence with PorGAs common in the MHI whereas PorTrm was more prevalent in the NWHI. This finding demonstrates the value of examining prevalence of individual diseases rather than combining diseases together. For example, if this study had combined all diseases together, then the average disease prevalence would be higher on the near-pristine reefs of the NWHI as compared to the impacted reefs of the MHI. However, this pattern is skewed by the high prevalence of a single disease, PorTrm, which has a lower prevalence in human impacted regions.

## Supporting Information

Figure S1 **Frequency of occurrence (FOC) and prevalence of different coral diseases among islands within the main Hawaiian Islands.** Standard error in parentheses. Por = *Porites*, Mont = *Montipora*, Trm = trematodiasis, GA = growth anomaly, TLS = tissue loss syndrome, MFTL = multi-focal tissue loss, Bl = bleaching, WS = white syndrome = tissue loss.
(DOC)

Figure S2 **Frequency of occurrence (FOC) and prevalence of different coral diseases among islands within the northwestern Hawaiian Islands.** Standard error in parentheses. Por = *Porites*, Mont = *Montipora*, Acro = *Acropora*, Trm = trematodiasis, GA = growth anomaly, TLS = tissue loss syndrome, MFTL = multi-focal tissue loss, WS = white syndrome = tissue loss, DTTS = discolored tissue thinning syndrome, BND = brown necrotizing disease, FFS = French Frigate Shoals, GAR = Gardner Pinnacles, PHR = Pearl and Hermes Atoll. ‘-‘ indicated that particular coral genera was not present within the transects.
(DOC)

Figure S3 **Average coral cover of dominant coral genera from surveys in the main and northwestern Hawaiian Islands.** Standard error in parentheses. FFS = French Frigate Shoals, GAR = Gardner Pinnacles, PHR = Pearl and Hermes Atoll. Coral cover determined by point-intercept method. ‘*’ indicates that coral was present within the belt transect but did not occur underneath the transect line so was not recorded by the point-intercept method.
(DOC)
