# Supplementary material for: Patterns of Coral Disease across the Hawaiian Archipelago: Relating Disease to Environment
Source: PLoS One. 2011 May 31;6(5):e20370. doi: 10.1371/journal.pone.0020370 (PMC3105043; doi:10.1371/journal.pone.0020370)
Supplement: Supplementary file 1 [file pone.0020370.s001.doc]

FOC (%)

**MHI**	Hawaii	Maui	Oahu	Kauai	Niihau	Lehua
	n=19	*n=12*	*n=27*	*n=12*	*n=6*	*n=3*
Por Trm	73.7	75	74.1	72.7	16.7	16.7
Por GA	47.4	91.7	74.1	63.6	0	0
Por TLS	5.3	25	40.7	18.2	0	0
Por MFTL	15.8	8.3	0	9.1	0	0
Por bl w/ TL	0	0	25.9	0	0	0
Mont GA	47.4	41.7	18.5	16.7	0	0
Mont MFTL	0	0	3.7	0	0	0
Mont WS	0	33.3	25.9	0	0	0

Average prevalence (%)

	Hawaii	Maui	Oahu	Kauai	Niihau	Lehua
Por Trm	1.4 (0.7)	2.8 (1.7)	0.77 (0.2)	0.58 (0.4)	0.03 (0.03)	0.04 (0.03)
Por GA	0.52 (0.2)	1.04 (0.2)	0.89 (0.4)	0.39 (0.2)	0	0
Por TLS	0.004 (0.004)	0.11 (0.5)	0.24 (0.09	0.05 (0.05)	0	0
Por MFTL	0.02 (0.01)	0.005 (0.005)	0.24 (0.09)	0.009 (0.009)	0	0
Por bl w/ TL	0	0	0.13 (0.09)	0	0	0
Mont GA	0.13 (0.07)	0.11 (0.05)	0.03 (0.02)	0.03 (0.03)	0	0
Mont MFTL	0	0	0.002 (0.002)	0	0	0
Mont WS	0	0.13 (0.08)	0.12 (0.06)	0	0	0
