# Supplementary material for: Patterns of Coral Disease across the Hawaiian Archipelago: Relating Disease to Environment
Source: PLoS One. 2011 May 31;6(5):e20370. doi: 10.1371/journal.pone.0020370 (PMC3105043; doi:10.1371/journal.pone.0020370)
Supplement: Supplementary file 2 [file pone.0020370.s002.doc]

FOC (%)

**NWHI**	FFS	GAR	Maro	Laysan	Lisianski	PHR	Midway	Kure
	n=11	*n=3*	*n=8*	*n=3*	*n=9*	*n=14*	*n=9*	*n=8*
Por Trm	72.7	33.3	100	100	100	70	71.4	57.1
Por GA	0	0	12.5	0	0	0	0	28.6
Por TLS	27.3	0	50	66.7	55.6	40	14.3	28.6
Por DTTS	18.2	0	12.5	66.7	55.6	10	14.3	0
Por BND	0	0	0	0	11.1	10	0	0
Mont GA	0	0	14.3	0	0	0	0	0
Mont MFTL	0	0	57.1	0	0	0	25	66.7
Mont WS	0	0	71.4	0	22.2	16.7	50	25
Acro WS	50	0	0	0	-	-	-	-
Acro GA	33.3	0	0	0	-	-	-	-

Average prevalence (%)

	FFS	GAR	Maro	Laysan	Lisianski	PHR	Midway	Kure
Por Trm	5.3 (3.7)	0.14 (0.14)	15.0 (3.0)	11.8 (4.0)	17.4 (5.5)	7.9 (5.9)	0.92 (0.64)	22.5 (11.7)
Por GA	0	0	0.03 (0.03)	0	0		0	2.6 (2.4)
Por TLS	0.9 (0.7)	0	1.3 (0.8)	0.26 (0.13)	1.5 (0.76)	0.14 (0.10)	0.13 (0.13)	1.6 (1.5)
Por DTTS	0.08 (0.06)	0	0.26 (0.26)	0.18 (0.09)	1.5 (0.66)	0.05 (0.05)	0.06 (0.07)	0
Por BND	0	0	0	0	0	0.01 (0.01)	0	0
Mont GA	0	0	0.05 (0.05)	0	0	0	0	0
Mont MFTL	0	0	1.6 (1.0)	0	0	0	0.29 (0.29)	0.6 (0.6)
Mont WS	0	0	0.4 (0.2)	0	0.15 (0.10)	0.05 (0.05)	0.55 (0.33)	0.19 (0.10)
Acro WS	1.1 (0.55)	0	0	0	-	-	-	-
Acro GA	0	0	0	0	-	-	-	-
