# Supplementary material for: Patterns of Coral Disease across the Hawaiian Archipelago: Relating Disease to Environment
Source: PLoS One. 2011 May 31;6(5):e20370. doi: 10.1371/journal.pone.0020370 (PMC3105043; doi:10.1371/journal.pone.0020370)
Supplement: Supplementary file 3 [file pone.0020370.s003.doc]

Average coral cover (%)

**MHI**	Hawaii *n=19*	Maui *n=12*	Oahu *n=27*	Kauai *n=12*	Niihau *n=6*	Lehua *n=3*		
Porites	15.3 (3.6)	12.7 (3.3)	14.7 (3.9)	3.2 (1.1)	0.17 (0.17)	0.33 (0.33)		
Montipora	3.2 (0.9)	20.8 (8.0)	7.8 (1.6)	2.9 (0.8)	1.7 (1.5)	0*		
Pocillopora	8.7 (1.8)	2.9 (1.2)	1.1 (0.4)	4.6 (1.4)	0.5 (0.5)	0*		
**overall coral cover**	**29.2 (3.2)**	**41.1 (7.5)**	**23.6 (3.9)**	**7.5 (1.8)**	**0.5 (0.5)**	**0.33 (0.33)**		
**NWHI**	FFS *n=11*	GAR *n=3*	Maro *n=8*	Laysan *n=3*	Lisianski *n=9*	PHR *n=14*	Midway *n=9*	Kure *n=8*
Porites	12.5 (3.6)	9.8 (3.9)	32.9 (5.3)	11.8 (3.4)	32.2 (7.7)	9.1 (5.8)	1.7 (0.6)	4.8 (3.2)
Montipora	0.9 (0.7)	0	6.1 (2.9)	4.6 (3.6)	5.2 (3.3)	1.7 (1.7)	6.8 (4.5)	1.1 (1.1)
Pocillopora	1.1 (0.7)	0	1.3 (0.9)	0	0.4 (0.3)	0.6 (0.4)	0.4 (0.3)	6.5 (3.0)
Acropora	8.6 (5.8)	2.0 (1.1)	0*	0*	0	0	0	0
**overall coral cover**	**24.0(5.5)**	**12.4 (4.7)**	**40.7(6.1)**	**16.4 (5.4)**	**38.3 (8.4)**	**11.9 (5.7)**	**9.2 (4.1)**	**12.8 (3.8)**
